# Integrating citizen engagement into evidence-informed health policy-making in eastern Europe and central Asia: scoping study and future research priorities

**DOI:** 10.1186/s12961-021-00808-9

**Published:** 2022-01-18

**Authors:** Bobby Macaulay, Marge Reinap, Michael G. Wilson, Tanja Kuchenmüller

**Affiliations:** 1 World Health Organization Regional Office for Europe, Copenhagen, Denmark; 2 McMaster Health Forum, McMaster University, Hamilton, ON Canada

**Keywords:** Citizen engagement, Evidence-informed policy-making, Health policy, Eastern Europe, Central Asia

## Abstract

**Background:**

The perspectives of citizens are an important and often overlooked source of evidence for informing health policy. Despite growing encouragement for its adoption, little is known regarding how citizen engagement may be integrated into evidence-informed health policy-making in low- and middle-income counties (LMICs) and newly democratic states (NDSs). We aimed to identify the factors and variables affecting the potential integration of citizen engagement into evidence-informed health policy-making in LMICs and NDSs and understand whether its implementation may require a different approach outside of high-income western democracies. Further, we assessed the context-specific considerations for the practical implementation of citizen engagement in one focus region—eastern Europe and central Asia.

**Methods:**

First, adopting a scoping review methodology, we conducted and updated searches of six electronic databases, as well as a comprehensive grey literature search, on citizen engagement in LMICs and NDSs, published before December 2019. We extracted insights about the approaches to citizen engagement, as well as implementation considerations (facilitators and barriers) and additional political factors, in developing an analysis framework. Second, we undertook exploratory methods to identify relevant literature on the socio-political environment of the focus region, before subjecting these sources to the same analysis framework.

**Results:**

Our searches identified 479 unique sources, of which 28 were adjudged to be relevant. The effective integration of citizen engagement within policy-making processes in LMICs and NDSs was found to be predominantly dependent upon the willingness and capacity of citizens and policy-makers. In the focus region, the implementation of citizen engagement within evidence-informed health policy-making is constrained by a lack of mutual trust between citizens and policy-makers. This is exacerbated by inadequate incentives and capacity for either side to engage.

**Conclusions:**

This research found no reason why citizen engagement could not adopt the same form in LMICs and NDSs as it does in high-income western democracies. However, it is recognized that certain political contexts may require additional support in developing and implementing citizen engagement, such as through trialling mechanisms at subnational scales. While specifically outlining the potential for citizen engagement, this study highlights the need for further research on its practical implementation.

## Background

Evidence-informed health policy-making (EIP) is “an approach to policy decisions that is intended to ensure that decision making is well-informed by the best available research evidence” [1] and implemented through systematic and transparent means. The types of evidence utilized, and how they are translated into policy, is a political decision with implications for a country’s health outcomes [2]. There are two types of evidence upon which health policy can be based, each comprising two subtypes: “explicit” knowledge consists of health data and systematic health research, while “tacit” knowledge comprises the beliefs and perspectives of policy-makers and citizens [3]. While adoption and institutionalization of evidence-informed approaches to health policy-making have increased globally, policy-makers tend to focus on the first three forms of evidence, neglecting the perspectives of citizens. Recently, WHO has actively promoted the inclusion of this form of evidence through mechanisms of citizen engagement (CE) [4].

### CE in EIP

The integration of CE in health policy-making is becoming increasingly prevalent, especially in western countries, recognizing the central importance of accountable and deliberative democracy in policy-making processes [5–7]. By harnessing the lay knowledge and social values of citizens, CE activities are perceived to improve the decision-making process and result in more effective public policies [8–11]. It is claimed that policies which contain a significant value-based element can be improved through the integration of citizens’ voices, illuminating factors which may not be clear to policy-makers [12, 13]. Within health policy, some consider CE a moral imperative, as the public represents both the funders and users of public health systems [14]. These characteristics of CE can be presented as three separate, but interrelated, aims [9, 15]:

- to enhance democratic engagement;
- to improve decision-making, resulting in more effective public policy;
- to develop the knowledge and capacity of citizens.

Numerous CE mechanisms have proliferated within health policy-making, with citizen juries and citizen panels being among the most widely adopted [16]. While these mechanisms differ in detail, almost all comprise a process which includes gathering a demographically representative group of citizens; presenting them with various forms of information on a topic; allowing them to discuss and deliberate, often involving the ability to question expert “witnesses”; and returning a final record of their conclusions [5, 6]. A review of CE in health policy-making suggested that while such mechanisms appear to have been successful in achieving the above aims, the “instrumental” effect of more effective public policy is less clear [17].

Throughout the coronavirus pandemic, citizens were largely absent from health policy-making, negatively affecting both the response to, and consequences of, the pandemic [18]. Involving citizens in the planning and implementation of interventions was considered crucial in encouraging the public to abide by new restrictions and make changes in their lives to curb the spread of the virus [19]. While it was acknowledged that decisions need to be made quickly and decisively (offered as a justification for not establishing a citizen platform to inform policy), this only added to calls for the institutionalization of CE within the process of evidence-informed health policy-making [18–20].

### Implementation of CE in EIP

The importance of taking a context-specific approach to the implementation of CE has been emphasized, suggesting that the selection of appropriate mechanisms may depend on a state’s social, economic and political characteristics [17, 21]. While CE in EIP proliferates in high-income countries (HICs) [11], less is known regarding its implementation in low- and middle-income countries (LMICs) and newly democratic states (NDSs). Despite significant recent developments (and publications) pertaining to evidence-informed policy-making in LMICs [22–31], the views of citizens are consistently absent from such discussions. A recently published scoping review of CE in EIP returned examples based exclusively in HICs, with over four in five of them being based in one country—Canada [16].

Of the literature which does exist pertaining to CE in EIP in LMICs, it is claimed that wider socio-economic inequalities and power imbalances necessitate a different approach to CE in EIP, specifically that public participation should not take place at the policy level [32]. While there is encouragement of, and belief in, participatory mechanisms to inform health policy from governments, nongovernmental organizations (NGOs) and supranational organizations [33], this participation tends to take place at the level of service providers, taking the form of coproduction and monitoring of local services [34]. This form of participation can increase citizens’ knowledge of the quality and choice of health provision they should expect (and their likelihood to demand it) [35] and may, as a result, improve health service delivery [36]. However, the lack of tangible power to influence policy leads to apathy, reducing citizens’ inclination to participate, and thus limiting the extent of these potential effects [33, 37].

Where public engagement has been integrated at the policy level in NDSs, “lay” citizens have been replaced by “representatives” claimed to speak on their behalf [38]. This does nothing to enhance democratic engagement or develop the knowledge and capacity of citizens, thus not achieving two of the three aims of CE. Disagreements between representatives further indicate a lack of settled understanding of citizen perspectives, raising doubts about how effective any resulting policy influence may be [38].

Justifications for not integrating CE in EIP centre on a policy-making culture which disregards the ability of citizens of LMICs and NDSs to participate and, subsequently, the value of any contribution derived through CE [37, 38]. However, no specific explanation is offered as to why this should be the case in LMICs and NDSs, but not in HICs. Indeed, as CE mechanisms in HICs incorporate the provision of sufficient information on a topic for a “lay” individual to be able to deliberate over it with others [5], is there any reason that this process cannot be similarly executed in LMICs and NDSs?

This research has two objectives. The first is to identify the factors and variables affecting the potential integration of CE in EIP in LMICs and NDSs. This will aim to provide an understanding of whether, and why, the implementation of CE in EIP may require a different approach outside of HICs. The second objective is to place this new understanding in context, and focus specifically on the potential to implement CE in EIP in one particular region. For reasons outlined in the subsequent section, this will focus on the “new democracies” of eastern Europe and central Asia (EE/CA) (where participatory and deliberative democratic innovations remain under-researched [39, 40]) to assess context-specific considerations for the implementation of CE in EIP.

## Methods

This research comprised two research methods. First, a reanalysis and updating of a scoping review of CE in EIP sought to source literature on CE in EIP in LMICs and NDSs. Second, a document analysis of literature pertaining specifically to political participation in EE/CA focused on the socio-political context of the region with regard to the development and implementation of CE. The methods adopted are detailed below.

### Scoping review

#### Identification

A recently published comprehensive scoping review of CE in EIP consisted exclusively of examples from HICs [16]. Adopting the same systematic search strategy, the scoping review was updated from its previous time threshold of April 2017 to include sources published up until December 2019 (Table 1). In addition, literature rejected from the final review was reassessed to consider whether any sources may be relevant to this research.

**Table 1:** Scoping review search strategy

Database name	Search strategy	No. hits
Ovid MEDLINE^®^ In-Process & Other Non-Indexed Citations, Ovid MEDLINE^®^ Daily and Ovid MEDLINE^®^ 14 April 2017 to 11 December 2019	1. (citizen* or patient* or public* or stakeholder* or deliberat*).mp 2. (panel* or jur* or deliberat* or conference* or dialogue* or poll* or map* or engag*).mp 3. (health* or "public health" or clinical).mp 4. Polic*.mp 5. 1 ADJ 2 6. 3 AND 4 AND 5	60
Embase 14 April 2017 to 11 December 2019	1. (citizen* or patient* or public* or stakeholder* or deliberat*).mp 2. (panel* or jur* or deliberat* or conference* or dialogue* or poll* or map* or engag*).mp 3. (health* or "public health" or clinical).mp 4. Polic*.mp 5. 1 ADJ 2 6. 3 AND 4 AND 5	121
Health Evidence 14 April 2017 to 11 December 2019	1. citizen* or patient* or public* or stakeholder* or deliberat* 2. panel* or jur* or deliberat* or conference* or dialogue* or poll* or map* or engag* 3. polic* 4. 1 AND 2 AND 3	20
Health Systems Evidence 14 April 2017 to 11 December 2019	Filters: Consumer participation in policy and organizational decisions, consumer participation in systems monitoring, consumer participation in service delivery	74
CINAHL 14 April 2017 to 11 December 2019	1. citizen* or patient* or public* or stakeholder* or deliberat* 2. panel* or jur* or deliberat* or conference* or dialogue* or poll* or map* or engag* 3. health* or "public health" or clinical 4. Polic* 5. 1 W1 2 6. 3 AND 4 AND 5	6
Cochrane Library 14 April 2017 to 11 December 2019	1. citizen* or patient* or public* or stakeholder* or deliberat* 2. panel* or jur* or deliberat* or conference* or dialogue* or poll* or map* or engag* 3. health* or "public health" or clinical 4. Polic* 5. 1 AND 2 AND 3 AND 4	21
Comprehensive search of included study reference lists, Open Grey, Grey Literature Report, and targeted websites 14 April 2017 to 11 December 2019	Similar search terms to those identified above were iteratively used to identify pertinent literature	8
		310

Furthermore, prominent academics conducting research in the fields of political science, public health, CE and development were requested to contribute literature relevant to the study aims. Individuals based in Brazil, Denmark and the United Kingdom were selected on the basis of their work in relevant fields.

#### Review of literature

**Fig. 1:**
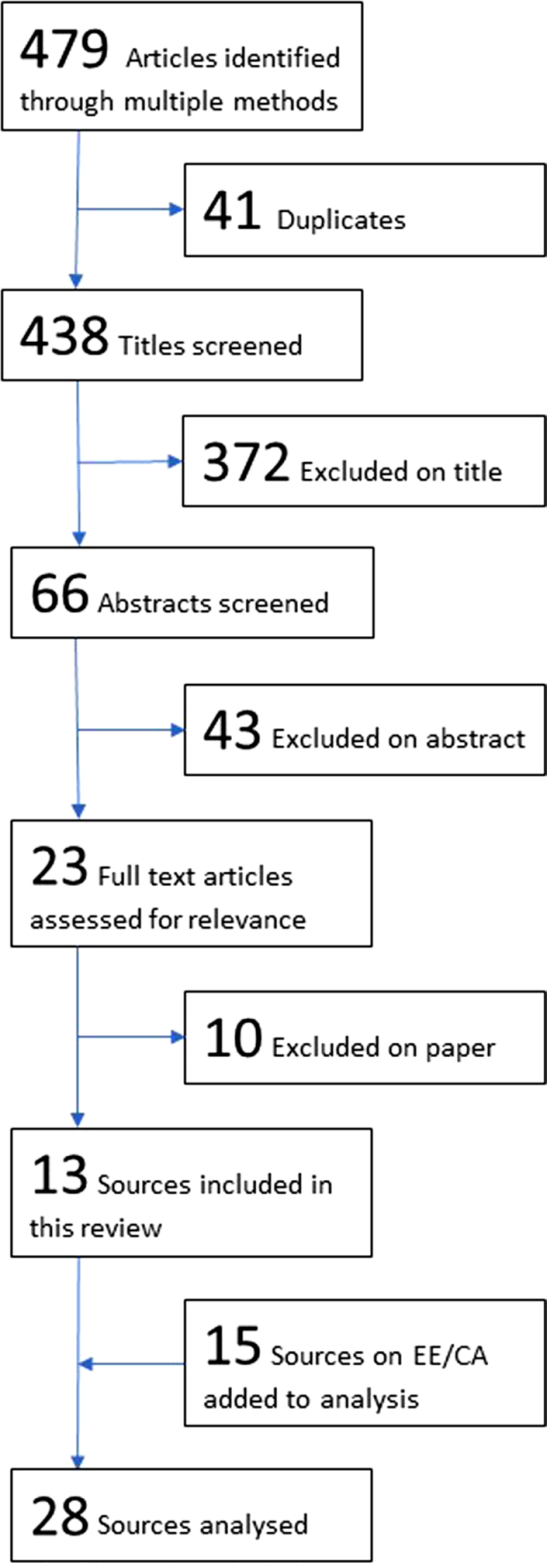
Flowchart of included articles

A staged process of assessing the potential relevance of search results considered title, abstract and full text in turn, rejecting sources at each stage if not relevant (Fig. 1). Inclusion criteria were threefold, requiring a focus on CE in EIP [16], LMICs or NDSs, and policy-making in health. Article screening and data extraction was led by one reviewer (BM) and verified by a second reviewer (TK) when eligibility was unclear.

#### Analysis framework and data extraction

Following the identification of relevant sources, data were coded thematically using QSR NVivo 12 software. Coding was conducted abductively, with all potentially relevant data coded at a granular level, before being considered against theoretical structures [41]. Granular subthemes were grouped into broader themes pertaining to the adoption of CE mechanisms, focusing on the following:

- nature of the CE mechanism;
- facilitators and barriers;
- impact on policy-making.

A theoretical framework which incorporated a wide range of contextual considerations related to CE in EIP, and especially in LMICs, was then selected as an appropriate approach to the structuring and synthesis of data [42]. This framework identified parameters through which it was possible to assess the potential adoption of CE in EIP in LMICs and NDSs—namely, the “willingness” and “capacity” of both policy-makers and citizens. “Willingness” refers to the likelihood that actors from the demand side (citizens) and supply side (policy-makers) will participate in CE mechanisms. “Capacity” concerns their ability, enabled or constrained by both internal and external factors, to do so. Additional considerations of “socio-political, economic, legal and other factors” [42] were integrated within this conceptual structure for the purposes of comparison. These variables provide a framework which could be adopted at a regional scale (described in the following section) or for more detailed case-based analysis, outlined below as a priority for future research.

### Literature review

#### Scope

In order to consider the practicalities of implementing CE in practice, we took a context-specific approach to considering the social, economic and political characteristics of one region [17, 21]. We considered it important for that region to have an established culture of EIP in order to consider the integration of CE specifically, as opposed to EIP more generally.

The WHO Evidence-informed Policy Network (EVIPNet) Europe is a knowledge translation (KT) network which promotes the systematic and transparent use of evidence in health policy-making [3, 43]. The network currently comprises 23 member countries, predominantly located in EE and CA. Building upon the work of EVIPNet, as well as its interest in the potential integration of CE within its remit, we focused on the EE/CA region, comprising both LMICs and NDSs.

#### Identification and analysis

While the above steps enabled us to develop an understanding of the factors pertinent to integrating CE into EIP in LMICs and NDSs, no literature was found pertaining to the EE/CA region. Thus, a secondary approach to sourcing literature on political culture, participation and democratic engagement in EE/CA was conducted through searches in research repositories and databases. This process aimed to consider the socio-political conditions of countries in the EE/CA region against the parameters defined through the first phase of analysis. Through sourcing of literature on the willingness and capacity of citizens and policy-makers in EE/CA, it is then possible to consider the potential application of CE in EIP in the region. These sources were coded using the same structure as outlined above and consider whether countries in this region reflect the conditions of LMICs and NDSs globally.

### Ethical clearance

As the data informing this study were sourced from secondary sources, ethical approval was not required, nor was it sought. The data that support the findings of this study are available from the corresponding author upon reasonable request.

## Results

### Sources

A total of 479 articles were identified through the initial literature search: 310 identified through the updated scoping review; 151 articles rejected by the previous scoping review of CE in EIP [16]; and 18 further sources provided by international colleagues (Fig. 1). Following the previously outlined process of assessing relevance, 13 articles were included in this research. The supplementary literature search added a further 15 articles relevant to political participation in EE/CA, leading to a final analysis of 28 articles. Details of these articles are listed in Table 2.

**Table 2:** Characteristics of included studies

	No. studies
Country/region focus	
Case study country	17
Global/regional focus	11
Publication status	
Peer-reviewed paper	26
Grey literature	2
Sourced through	
Rejected papers from previous scoping review [16]	3
Updated scoping review	7
Suggested by international colleagues	3
Non-systematic search for literature on political participation in EE/CA	15

The following sections detail findings in relation to CE in EIP in LMICs and NDSs, and subsequently focusing specifically on the EE/CA region. Findings are structured in line with conceptual considerations for the integration of CE in LMICs and NDSs [42], outlined previously. The structuring of findings into the “willingness” and “capacity” of both policy-makers and citizens illustrates relevant contextual considerations and opportunities for integrating CE within EIP, first in LMICs and NDSs, and then more specifically in EE/CA. Table 3 summarizes the main elements of each theme.

**Table 3:** Summary of findings

	LMICs and NDSs	EE/CA
Willingness		
Policy-makers	Lack trust in citizenry. Dependent on political gain. Remains underfunded	Increasing, but largely dependent on political gain. Can be developed through financial incentives and deliberative mechanisms
Citizens	Lack belief in impact of engagement. Requires broad remit, transparency and remuneration	Generally low, due to political distrust. Increases when tangible results become apparent, such as in local policy and service provision
Capacity		
Policy-makers	Requires funding and broader political commitment	Capacity is apparent, evidenced by existing participatory mechanisms
Citizens	Requires support and capacity-building. Best suited to moral or ethical judgements	Low, but increasing across the region. Evidence of sufficient capacity if adequate support provided

### CE in EIP in LMICs and NDSs

#### Willingness of policy-makers

CE in EIP ultimately represents a devolution of power from policy-makers to citizens [44]. Willingness, in this regard, requires a level of trust that such authority will be responsibly wielded or, from a partisan perspective, that it can still serve certain political interests [42, 45]. Competitive political environments may therefore make CE more prevalent, but may also be biased and politically motivated from the outset [42], resulting in the failure to achieve more effective policy [46] or the representation of citizens’ voices [44, 47].

The willingness of policy-makers to implement and respond to CE mechanisms legitimizes the process, improving outcomes and increasing citizen participation [42, 44–49]. Support is claimed to be growing among policy-makers for the integration of CE into health policy-making at the national and subnational levels in LMICs [50], although many CE mechanisms continue to be underfunded and ad hoc in nature [51].

#### Willingness of citizens

Three interrelated factors were found to affect the willingness of citizens in LMICs and NDSs to engage in CE mechanisms: authority, information and opportunity cost. First, there was little belief that engagement would make a positive or material difference in the lives of individuals or communities [42, 51, 52]. This was due to a lack of genuine devolution of authority, as well as political inefficiencies and corrupt practices reducing trust in the entire political system [45, 47]. Conversely, levels of participation were reported to improve when citizens were assured of a wide remit and authority, and kept informed as to the impact of their participation [44, 51, 53].

Second, transparent information pertaining to the subject and process of the CE mechanism improved trust and participation [44, 45, 48, 52]. This included having the ability to see how such engagement had been considered by policy-makers, and the extent to which it had influenced subsequent policy decisions. Such information could be provided through government transparency initiatives or an independent and competitive media [42, 51].

Finally, for many citizens in LMICs, participation in such initiatives is not considered a priority, due to more pressing matters of work and family [44, 53]. This was further exacerbated where engagement was considered to have little material impact, either through disregard or poor communication on the part of policy-makers, relating to the previous two points. In this regard, financial incentives [49] and limited time commitment [51] overcame some of this unwillingness.

#### Capacity of policy-makers

The capacity of policy-makers to implement CE mechanisms depends on a number of factors including increased training, resourcing and capacity-building of government departments tasked with organizing and responding to CE mechanisms [42, 44]. CE is “labour-, cost- and time-intensive” [52], with sometimes unclear results, and may not be the top priority for health policy-makers in LMICs and NDSs [46].

International agencies can advocate for the integration of CE mechanisms, providing funding and support for their mainstreaming within decision-making processes [49]. While this could enhance the capacity of policy-makers to integrate CE in EIP, it is cautioned that such agencies can also seek to influence policy themselves, thus potentially removing even more influence from citizens [42, 54].

#### Capacity of citizens

There were concerns regarding the ability of citizens to comprehend the subject matter and actively engage in the CE process [44, 46]. However, while citizens may lack experience in this environment, the provision of support, training and technical assistance is considered to equip them with the ability to adequately assess information and derive conclusions from it [48, 49, 53]. NGOs and other civil society organizations can support populations in contexts where citizens have no experience of engaging with the public sector [42, 44, 47, 51].

Furthermore, structuring CE activities to seek citizen input in the form of moral or ethical judgements was considered to overcome any such barriers by not necessitating high levels of technical knowledge among the citizenry [54, 55]. CE mechanisms can seek to elicit such judgements and “lay” perspectives to complement other forms of evidence contributing to decision-making, for example in the potential social acceptability of a planned public health intervention.

### CE in EE/CA

While large and culturally heterogeneous, much of the EE/CA region shares a common political legacy which influences the relationship between citizens and the state [39, 42]. Most countries in the region are officially considered democracies; however, some are described as “hybrid regimes” [56] due to the curtailing of certain freedoms usually expected in modern democratic societies [39, 57].

#### Willingness of policy-makers

As in LMICs and NDSs more broadly, the willingness of policy-makers to cede power to citizens will depend upon their incentive to do so. One such incentive may be the encouragement of donors and international organizations (such as the World Bank or United Nations (UN) agencies) to find means through which citizens can engage in policy-making [42, 58], possibly carrying financial implications for a country. A range of direct democratic innovations in the region imply that this may be occurring, with referendums being the most common means of engaging the electorate. However, the introduction of democratic mechanisms must be matched with commensurate encouragement of a participatory political culture, to ensure an enhancement of the engagement and influence of citizens [39, 59, 60]. For example, political actors have been accused of the “colonization” [57] or “hijacking” [61] of democratic exercises, resulting in a lack of genuine improvements to CE [60]. These experiences have delegitimized both the process and the result of these mechanisms, reducing public participation and political trust [57, 62].

Conversely, participatory mechanisms which included a deliberative element were claimed to lead to “rational and just” decisions, which will “be accepted as politically legitimate” [56, 62] by both citizens and policy-makers. Furthermore, where deliberation was included in CE activities, it led to numerous other forms of civic engagement [63] and was considered key in building trust in the political system and between citizens [56, 60].

#### Willingness of citizens

Democratic engagement is lower in EE/CA than in western Europe, attributed in the literature to distrust of the political system [64, 65]. While historically this was due to citizens being “estranged” from power [40, 56, 63], subversion of democratic mechanisms by political actors continues to damage trust in policy-makers [39, 57, 59–61], and subsequently to reduce citizens’ willingness to participate [63, 66]. This does not, however, imply an ambivalent citizenry, with examples of informal political actions occurring in the region even prior to democratization [40, 67]. Contemporary acts such as boycotts of referenda to delegitimize or invalidate the outcome have tangible results [60, 61, 66]. Similarly, participation in formal political processes increases following a tangible change brought about by CE [58].

Public services are often the most immediate and visible representation of the tangible impact the state can have on people’s lives. As a result, local politicians and political institutions are often considered more credible and trustworthy than those at a national level [58, 66], with their actions having a greater impact upon the lives of local people. As was evidenced in numerous examples, engaging citizens in discussions around local policy, and seeing the effects of that engagement on local service delivery, can build trust in democratic systems and lay the foundations for CE mechanisms at the national scale [39, 56, 67, 68].

#### Capacity of policy-makers

The proliferation of participatory innovations in EE/CA indicates that policy-makers have the capacity to provide opportunities for engagement [39, 40]. However, while the effective implementation of CE mechanisms depends on maintaining political neutrality and genuine devolution of authority [60], policy-makers’ motivations for supporting CE may not. In this sense, the capacity of policy-makers to facilitate a participatory mechanism does not necessarily lead to CE in EIP, due to a lack of willingness to do so without political bias [56]. Thus, this capacity is rendered moot in the absence of faith in the objectives of CE, which in itself is a manifestation of the lack of political trust.

#### Capacity of citizens

The literature indicates low levels of political education in the EE/CA region which can manifest in the dominance of, and reverence to, political parties and other civil society actors [60, 61], doing little to empower individual citizens [42, 56]. However, citizens of states “with little or no democratic experience may be more perceptive about politics and democracy than is often assumed” [64]. Where education and training is provided to citizens, the result is a greater understanding of the implications of their engagement, and the influence that their participation has on policy [58, 68]. Such training has increased the participation of citizens in democratic mechanisms [56], weakened partisan affiliation [60, 61] and increased the capacity of citizens to engage in complex deliberations around constitutional design [57, 62]. In such instances, there was evidence that participants comprehended the subtleties and interconnections of local, national and international power politics, and how to influence them [58, 66].

## Discussion

### Principal findings

This study found that the contextual and conceptual factors pertinent to consider when integrating CE into EIP in LMICs and NDSs related to the willingness and capacity of both citizens and policy-makers to engage in the process. Therefore, the claim that a different approach to CE was required in LMICs [21] was found not to be due to a country’s financial capabilities (as this labels implies), but in their political culture [32]. Specifically, sufficient trust between citizens and policy-makers was identified as a necessary prerequisite to develop the willingness of each to faithfully engage in CE mechanisms and avoid negative effects upon democratization and health outcomes [46].

Political trust relates to the willingness of both citizens and policy-makers to engage in CE mechanisms, fulfilling two of the three aims of CE [9, 15]—enhancing democratic engagement (citizens) and improving decision-making (policy-makers). The above results reflected previous research outlining how subnational and service-level engagement with citizens can enhance communication and trust between citizens and policy-makers [33]. This local engagement can then build the foundations of capacity, willingness and mutual trust for policy-level engagement with citizens [39, 56, 67, 68]. However, recognition is made of the fact that, regardless of its benevolent aims, CE represents a ceding of power from policy-makers [32, 44]. A policy-making culture which does not value citizen input and is restricted in both capacity and finances may therefore be unwilling to favour such engagement [37].

Furthermore, this research sought to understand context-specific considerations for the implementation of CE in EIP in EE/CA. Findings indicate that political trust is low in the EE/CA region, due largely to a legacy of political alienation [40, 56, 63]. Once a sufficient level of political trust is established, there is no indication that CE mechanisms would require a different approach from that taken in HICs [5, 16]. However, a further prerequisite step may be required in building the knowledge and capacity of citizens [9, 15]. The successful experience of doing so counters claims that the citizens of NDSs are incapable of participating in CE due to their lack of knowledge, ability or desire [38, 46, 64].

This study offers suggested means of developing the political culture and capacity of countries to facilitate the adoption of CE in EIP. It is hoped that these suggestions will illustrate the benefits of engagement in CE to both citizens and policy-makers, encouraging their future engagement and forming the basis of ongoing efforts to develop a strategy for the implementation of CE in EIP in EE/CA. The first three relate to the building of political trust through the design, undertaking and reporting of CE mechanisms. These will help to outline the benefits of engagement to citizens, illustrating their ability to understand and engage in policy discussions and effect real change within their own lives and circumstances. The fourth pertains to the role played by international organizations in facilitating increased engagement of both citizens and policy-makers. This can serve as a financial or diplomatic incentive for policy-makers to integrate CE, as well as providing a trusted intermediary through which to better understand how CE can improve policy-making processes.

1. Conducting CE at decentralized levels was claimed to increase trust in political systems and the ability of citizens to engage in discussions [42, 44, 45, 47, 48, 50, 53]. Therefore, it is recommended that such subnational mechanisms be implemented within health service delivery and other decentralized public sector provision. Subsequent CE activities can be scaled up to national policy-making in EE/CA [21, 39, 56, 67, 68].
2. Once a baseline of political trust has been established, the practical undertaking of the CE mechanism can build upon it through the provision of information and the ability to deliberate over it [32, 57, 62]. Such information can take the form of written documents and the ability to question a wide range of stakeholders knowledgeable on the topic of deliberation. These steps represent the central elements of CE mechanisms such as citizen panels and citizen juries [5, 16].
3. The policy-making process must be transparent—that is, the manner in which this form of evidence is utilized and weighed against others must be openly communicated both to participants in CE mechanisms and the broader citizenry [44, 51, 53]. This will confer legitimacy and accountability upon the process, evidence the tangible power held by citizens and consequently increase willingness to participate in future engagement exercises [7, 32, 35].
4. International organizations such as the UN and World Bank can encourage and support policy-makers in adopting CE mechanisms. This can either be by advocating for the contribution it can make to effective policy-making, or by making financial or other support dependent upon the integration of CE [37]. Furthermore, the capacity of citizens to engage in CE mechanisms can be built through the provision of education and training by such non-state actors, as well as local civil society organizations [42, 44, 47, 51, 54, 56, 58, 68].

### Strengths and limitations

The main strength of this study, the first of its kind conducted in the region, is in its comprehensive methodological approach to sourcing and analysing relevant literature. The triangulation of scoping review methodology with other literature review approaches led to a substantial volume of data being assessed and analysed. Through doing so, this study has outlined the large gap in knowledge around this topic and laid the foundations for future research and practical application.

The main limitation of the study lies in the lack of a second reviewer of literature. This may have led to unconscious bias in the selection and analysis of sources, especially considering the exploratory, non-systematic nature of the review of literature pertaining to the EE/CA region. Furthermore, the exclusion of non-English-language resources may have limited what materials could be sourced. The inclusion of Russian-language resources may have expanded the literature base around this topic, and could form the basis of a follow-up study.

### Implications for practice

**Fig. 2:**
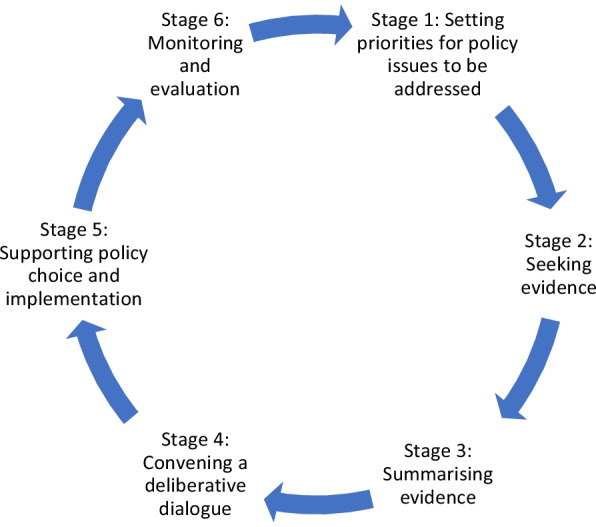
EVIPNet Europe policy action cycle

EVIPNet Europe is one of a number of initiatives which seeks the requisite understanding of what works, and in what contexts, to be in a position to advocate for the adoption of CE within EIP. EVIPNet supports the establishment of KT platforms (KTPs) in WHO Member States to foster closer relationships between researchers and policy stakeholders, leading to the institutionalization of evidence-informed policy-making [30, 69]. KTPs follow a “policy action cycle” (Fig. 2) through which its members can undertake the practical steps involved in EIP [3]. CE can be incorporated into this process, integrating the tacit knowledge of citizens alongside the three other forms of evidence with which to inform policy [43, 70].

The potential for CE to be successfully implemented outside of HICs, subject to certain processes, should encourage EVIPNet Europe to pursue further efforts in the region. This may come in the form of opportunities for scaling up subnational CE activities by building on existing networks and practices. EVIPNet Europe must take these opportunities into account when supporting the expansion and operationalization of CE in EIP in EE/CA [3, 70].

### Implications for research

While this research has established a baseline understanding of the field of literature and conceptual possibilities, further research is necessary before EVIPNet Europe can develop a strategy to integrate CE into its policy action cycle [3, 4, 70]. Specifically, in building upon these conclusions and the stated importance of acknowledging context-specific considerations [46], it is suggested that future research adopt a case-based approach to assessing individual countries in the region. This would be guided by the socio-political, economic, legal and other factors considered to affect adoption and outcomes of CE [42], as well as potential “entry points” for CE mechanisms within the health system, and at different stages of the policy action cycle [3, 16, 70].

Subsequently, a typology of countries can be developed, differentiating between countries within the region, as well as EVIPNet Europe members outside of EE/CA. A central aspect of this typology will be an assessment of the sufficiency of political trust necessary for conducting successful CE in EIP, and how that may be measured. Longer-term monitoring and evaluation of CE in EE/CA can shed light on the impact of their adoption and whether they achieve the aims of CE [15].

## Conclusions

This research reveals a symbiotic and mutually reinforcing relationship between political trust and democratic participation in the form of CE. While CE aims to enhance and improve democratic participation, effective policy-making and political education, each of these must also be developed prior to its implementation at the national scale. This can be achieved through the four steps outlined above. Only then can a sufficient level of political trust be achieved to meet the aims of CE in EIP: (further) enhancement of democratic engagement; improvement in decision-making (based on a broader evidence-base); and the development of the knowledge and capacity of citizens [15].

The findings of this study suggest that CE can be gradually developed within local/decentralized decision-making spaces, which already possess greater levels of public trust. By expanding these mechanisms, and in collaboration with international organizations and local civil society, the capacity and willingness for both citizens and policy-makers to engage will be gradually developed. CE mechanisms can then be executed in a manner which involves educating citizens on a topic and allowing them to deliberate before returning a judgement. If this exercise holds genuine authority and the resultant policy change is transparently communicated to participants and the wider citizenry, this will further increase political trust and enable future CE activities. This virtuous cycle of political trust has implications far beyond EIP, and may contribute to the ongoing democratization of the EE/CA region.

Such efforts may not come in time to integrate the voices of citizens into the response to the coronavirus pandemic. In such circumstances, and with limited public funds, CE may not be considered a political priority [47]. However, the institutionalization of CE in EIP will allow for citizens’ voices to contribute to designing effective policies to combat future health crises, while building stronger and more trusting societies [18–20]. The need for collaboration between citizens and policy-makers to ensure the survival of each has never been more apparent.

## Data Availability

The datasets used and/or analysed during the current study are available from the corresponding author on reasonable request.
